# Extracellular Biosynthesis, Characterization and Antimicrobial Activity of Silver Nanoparticles Synthesized by Filamentous Fungi

**DOI:** 10.3390/jof10110798

**Published:** 2024-11-16

**Authors:** Iuliana Răut, Mariana Constantin, Raluca Șuică-Bunghez, Cristina Firincă, Elvira Alexandrescu, Ioana Cătălina Gîfu, Mihaela Doni, Lucian-Gabriel Zamfir, Ana-Maria Gurban, Luiza Jecu

**Affiliations:** 1 Biotechnology and Bioanalysis Department, National Institute for Research & Development in Chemistry and Petrochemistry—ICECHIM, 202 Independentei Spl., 060021 Bucharest, Romania; iulia_rt@yahoo.com (I.R.); marriconstantin@yahoo.com (M.C.); raluca_bunghez@yahoo.com (R.Ș.-B.); firincacristina@yahoo.com (C.F.); elviraalexandrescu@yahoo.com (E.A.); gifu_ioanacatalina@yahoo.com (I.C.G.); mihaela.doni@icechim.ro (M.D.); lucian.zamfir@icechim.ro (L.-G.Z.); 2 Faculty of Pharmacy, Titu Maiorescu University, 16 Bd. Gh. Sincai, 040441 Bucharest, Romania; 3 Faculty of Biology, University of Bucharest, Splaiul Independentei 91–95, 050095 Bucharest, Romania

**Keywords:** filamentous fungi, green chemistry, silver nanoparticles, *Cladosporium cladosporoides*, antimicrobial activity

## Abstract

The green synthesis of metal nanoparticles has received substantial attention due to their applications in various domains. The aim of the study was to obtain silver nanoparticles (AgNPs) by green synthesis with filamentous fungi, such as *Cladosporium cladosporoides*, *Penicillium chrysogenum*, and *Purpureocillium lilacinum*. Fungal species were grown on nutrient media and aqueous mycelium extracts were used to reduce Ag^+^ to Ag (0). The silver nanoparticles were analyzed by various techniques, such as UV-Visible spectroscopy (UV-Vis), scanning electron microscopy (SEM), Fourier transform infrared spectroscopy (FTIR), transmission electron microscopy (TEM), dynamic light scattering (DLS), and Zeta potential. The formation of silver nanoparticles was confirmed by UV-Vis spectroscopy and the color change of the mixture containing metal precursor and aqueous mycelium extract. FTIR displayed different functional groups as capping and reducing agents for the biosynthesis of AgNPs. SEM and TEM provided information on the particles’ morphology. DLS diagrams indicated mean particle diameters in the 124–168 nm region. All biosynthesized AgNPs had negative zeta values, which is a sign of good stability. Silver nanoparticles were evaluated for antimicrobial activity, and the most active were those synthesized with metabolites from *Cladosporium*, leading to 93.75% inhibition of *Staphylococcus aureus*, 67.20% of *Escherichia coli*, and 69.56% of *Candida albicans*. With the highest microbial inhibition percentage and a very good Poly Dispersion Index (Pd I), *Cladosporium cladosporoides* was selected as an environmentally friendly source of silver nanoparticles that could be used as a potential antimicrobial agent.

## 1. Introduction

Silver nanoparticles have been applied in multiple ways in various fields, such as in wastewater treatment [1,2,3,4], in agriculture for plant growth and development [5,6,7], as functional food additives [8,9], as antimicrobial agents [10,11,12,13,14], in biocatalysis [15,16], and in the design and development of (bio)sensors [1,2,3,4,5,6,7,8,9,10,11,12,13,14,15,16,17,18]. The synthesis of metal nanoparticles can be achieved by reducing metal ions into uncharged nanoparticles using a different type of reducing agents by two methods, namely, top-down and bottom-up. The first method is a destructive one, involving the breakdown of the larger aggregates into smaller units, which are then converted into nanoparticles ranging in size from 20 nm to 100 nm. The bottom-up technique involves the formation of nanoparticles through the growth and self-assembly of atomic and molecular aggregates. Each technique has advantages and disadvantages, with the way forward being chosen depending on the primary materials, potential applications, and economic aspects [19,20].

Nanoparticles can be obtained through two types of mechanism, intracellular and extracellular. The extracellular way is preferred since the process takes place in a solution created through the interaction of metal ions and microbial enzymes, mainly reductase, from an aqueous extract of mycelium. It is an easier way, without the step of separating nanoparticles from hyphae, where sometimes the use of ultrasound or appropriate detergents is necessary to separate nanoparticles attached to the cell wall. The use of whole-cell microorganisms has limitations, including the binding of nanoparticles to cellular components and potential product loss.

The green synthesis of nanoparticles based on microorganisms and plants is a new approach which is friendly to and safe for the environment, characterized by a reduction in energy and resource consumption, which is being preferred to conventional chemical and physical methods [21,22]. The biogenic process is simple and easily reproducible, allowing nanoparticles to be obtained with desired characteristics that are specific to the targeted applications. Biological systems produce various biochemicals, such as flavonoids, terpenoids, alkaloids, and polyphenols, which act as reducing and stabilizing agents. According to the literature reviewed by Chopra et al. [23], the biogenetic metal nanoparticles are more active than those physical/chemically synthesized.

Several microorganisms, mainly bacteria and fungi, are used in the synthesis of metal nanoparticles, due to their enzymatic activity and metabolic processes, by extracellular or intracellular routes. The properties of biosynthesized nanoparticles depend on many factors, such as the characteristics of the microorganism (e.g., genus, specie), the extracts and metabolites used, the metal precursor and its concentration, the composition of the medium for microbial cultivation, and the parameters of the biosynthesis process (e.g., speed of stirring, incubation, temperature, pH). All these factors control the process, and thus impose the morphology, shape, size, and topography of the nanoparticle surface, which together determine future applications.

The first group of microorganisms used in nanoparticle biogenesis research was bacteria. Bacterial species are known for their rapid growth and relative simplicity of cultivation and manipulation, and many species such as *Bacillus* sp., *Pseudomonas stutzeri*, *Proteus mirablis*, *Vibrio alginolyticus*, and *Streptomyces* sp. have been used to obtain nanoparticles, mainly by the extracellular route, which is simpler [24,25,26]. Several possible problems in the use of bacteria have been identified, such as slow synthesis speed, the easy contamination of the culture, the specific morphology of bacterial cells with a low level of proteins responsible for the reduction of metal ions compared to fungi, and limited control for obtaining the size of nanoparticles [27].

Fungi are important candidates for the manufacturing of metal nanoparticles, since they secrete a wide variety of biomolecules, including proteins, enzymes, and extracellular metabolites, that would increase the productivity of nanoparticle synthesis, producing a large amount of biomass by easily growing on cheap and large available nutrients. These molecules stabilize biogenic nanoparticles and contribute to their specific physico-chemical properties. Based on the analysis of the specialized literature, numerous studies have been published on the synthesis of metal nanoparticles mediated by fungi [28,29,30,31,32,33]. The conversion of precursor metal salts into nanoparticles is a faster process, leading to the formation of particles with a large surface area due to the presence of the macromolecules secreted by mycelia. Despite the greater resistance to metal precursor toxicity exhibited by fungi compared to bacterial species, in some cases, the concentration of the precursor could be a limiting factor that inhibits microbial growth [34].

Several filamentous fungi belonging to various genera, including *Aspergillus* [35,36], *Cladosporium* [37,38], *Penicillium* [39,40], *Fusarium* [41], *Paecilomyces* [42], *Trichoderma* [43,44], and *Verticillium* [45], etc., have been reported for the biosynthesis of nanoparticles. Also of great interest were *Basidiomycetes* from different taxonomic groups, such as, *Ganoderma lucidum* and *Ganoderma sessiliforme*; *Lentinus edodes*; *Agaricus bisporus*; *Phaenerochaete chrysosporium*; *Pleurotus ostreatus* and *Pleurotus sajor-caju*; and *Phellinus adamantinus* [46,47]. Most of these species used for the preparation of nanoparticles are edible and medicinal mushrooms, which produce compounds that exert anti-inflammatory, anticancer, antioxidant and antimicrobial activities. The ability of fungi to be involved in the synthesis of metal nanoparticles contributes to the rapid development of the so-called myco-nanotechnology, producing nanoparticles characterized by reactivity and bioavailability adapted for different applications.

The list of metal nanoparticles synthesized by fungi is very large, including various elements, such as silver, gold, selenium, tellurium, platinum, palladium, etc. Our attention was focused on silver, because it has antiseptic and antimicrobial action in all the forms in which it is found. The development of nanotechnology and the growing interest in finding antimicrobial agents capable of inhibiting multidrug-resistant microbes have focused research on silver nanoparticles that express effective antimicrobial power without toxicity to human cells.

A recent study highlighted the significant activity of silver nanoparticles (AgNPs) as a defensive barrier for most pathogens, killing approximately 650 types of disease-causing microbes [48].

The conditions of cultivation and the characteristics of species within the genus determine the synthesis of different metabolites and proteins that are involved in the biosynthesis process. The microorganism used in the biosynthesis of metallic nanoparticles represents the significant starting point for the high-yield biosynthesis of particles with the desired features.

Therefore, we explored the potential of several fungi for the biogenic synthesis of silver nanoparticles as a further extension of our previous research that demonstrated the biosynthesis of nanoparticles using *Ganoderma lucidum* [49]. The main purpose of this study was to screen filamentous fungal species for the efficient synthesis of AgNPs. The biogenic nanoparticles were characterized with various analytical techniques, such as UV-visible spectroscopy, scanning electron microscopy (SEM), Fourier transform infrared spectroscopy (FTIR), transmission electron microscopy (TEM), dynamic light scattering (DLS), and zeta potential. The antimicrobial action of biosynthesized silver nanoparticles was evaluated with the following microbial species: a Gram-positive bacterium, *Staphylococcus aureus*; a Gram-negative bacterium, such as *Escherichia coli*; and a yeast (type of fungi), such as *Candida albicans*. The comparison of the results obtained from the evaluation of the antimicrobial activity allowed the selection of a fungal isolate that can be useful for various medical and pharmaceutical applications.

## 2. Materials and Methods

### 2.1. Fungal Isolates and Mycelial Growth

The following fungal strains belonging to the Microbial Collection from the National Institute for Research and Development in Chemistry and Petrochemistry-ICECHIM, *Cladosporium cladosporoides*, *Penicillium chrysogenum*, and *Purpureocillium lilacinum*, were used in the present study. The strains were maintained on a potato-dextrose-agar medium, (PDA) containing (g/L): 4.0, peptone (Scharlau, Scharlab S.L., Barcelona, Spain); 20, glucose (Scharlau, Scharlab S.L. Spain). Actively growing mycelia (1 piece of 5 mm diameter) were grown from the new prepared stock cultures on a PDB liquid medium (Scharlau, Scharlab S.L. Spain), having the following composition (g/L): 4, potato peptone; 20, glucose. The Erlemnayer flasks of 250 mL, containing a 100 mL medium, were incubated in a Heidolph UNIMAX, (Schwabach, Germany) rotary shaker in dark, at 28 °C and 140 rpm for 7 days. Subsequently, the mycelia were collected after filtration, and washed 3–4 times using sterile double-distilled water to remove the remaining components of the culture medium. Then, 10 g of weighed mycelium was suspended in 100 mL of sterile distilled water and incubated at 28 °C for 3 days in the dark, under stirring at 150 rpm.

### 2.2. Preparation of AgNPs

AgNPs were synthesized according to the previously adopted experimental protocol [49]. Briefly, aqueous fungal extracts were used for the synthesis of AgNPs, namely, 40 mL of filtrate was mixed with 20 mL of 0.1 mol/L AgNO_3_ solution (Carlo Erba, Reagents GmbH, Emmendingen, Germany). The reaction mixtures were incubated, and the resulting mixtures were evaluated for color change, a sign of nanoparticles synthesis. The Erlenmeyer flasks with mycelia only and without AgNO_3_ were used as control. The reaction mixtures were incubated in a rotary shaker at 25 °C and 150 rpm in the dark, for 5 days. All experiments were done in triplicate. The color change of each reaction liquid was observed. The obtained AgNPs colloidal suspensions were recovered by centrifugation.

### 2.3. Characterisation of the Biogenic Nanoparticles

The biogenic silver nanoparticles were characterized using standard techniques, namely, UV-Vis spectroscopy, scanning electron microscopy (SEM), and FTIR analysis under the conditions described in previous work [49]. Thus, the reduced silver ions were analyzed in the range of 250–650 nm on the Cintra 202 UV-VIS spectrophotometer. The spectral characterization of the biosynthesized AgNPs was recorded by Fourier transform infrared spectroscopy (FTIR) on a Perkin Elmer FTIR, in the range of 600–4000 cm^−1^, at room temperature, in standard 10 mm quartz cuvettes, and with ultrapure water as a blank. Evaluation of the nanoparticles’ surface morphology was performed using an FEI Quanta 200 Scanning Electron Microscope, with a low vacuum mode (chamber pressure of 133 Pa) and an acceleration voltage of 30 KV. The specimens were coated with a 5 nm layer of gold using a Quorum 150R ES Plus Sputter Coater. AgNPs were also analyzed by transmission electron microscopy (TEM). 5 µL of each sample were placed on a type 1811 carbon film 200-mesh copper grid (TedPella, Redding, CA, USA). The grids were then gently wiped. After drying, the grids were analyzed at an accelerating voltage of 200 kV using a Tecnai F20 G^2^ TWIN Cryo-TEM (Thermo Fischer Scientific, Waltham, MA, USA). The particle sizes of the silver nanoparticle solution were assessed by dynamic light scattering (DLS) on a Nano Zetasizer ZS (Malvern Instruments, Malvern, UK). Zeta potential (ξ) measurements were performed at room temperature. The values for the electrokinetic potential (ξ) were determined using the Helmholtz-Smoluchowski equation. All tests were run three times, and the results were averaged by number/intensity/volume.

### 2.4. Antimicrobial Activity

The antimicrobial efficiency of biogenic AgNPs was investigated with the agar diffusion method against a selection of medically relevant strains: *Staphylococcus aureus* (ATTC 25932); *Escherichia coli* (ATTC 25922); and *Candida albicans* (ATTC 10231). The test procedure and conditions were the same as described in previous work [49]. Briefly, the bacterial strains were cultured onto Mueller–Hinton agar (Scharlau, Barcelona, Spain), and the yeast strain was cultured onto Sabouraud agar (Scharlau, Spain). The plates were swabbed with the fresh inoculum and a volume of 50 µL AgNPs suspension was distributed onto a sterile disk. Subsequently, positive controls represented by antibiotics, and negative controls represented by the tested strains grown in normal conditions were prepared. The following antibiotics were used for positive controls: clindamycin (2 µg) against *S. aureus*; norfloxacin (10 µg) against *E. coli*; and ketoconazole (50 µg) against *C. albicans*. The plates were incubated for 24 h at the appropriate temperature and the antimicrobial activity was evaluated by measuring the diameter of the inhibition zones. Microbial growth inhibition was calculated using formula [38]:

% inhibition = (A_control_ − B_sample_)/A_control_ × 100 (1)

where A_control_ = growth diameter in the control plates, B_sample_ = growth diameter in the treated plates.

### 2.5. Minimum Inhibitory Concentration MIC Determination

Minimum inhibitory concentration (MIC) is defined as the lowest concentration of an antimicrobial agent showing no visible bacterial growth after incubation time. Minimum inhibitory concentration was determined by broth serial dilutions [40,41] on a 96-well microtiter plate, in Mueller–Hinton Broth for bacteria and Sabouraud Dextrose Broth (SDB) for fungi. A volume of 150 µL of culture medium was distributed in each well of the microplate. For the MIC test, aliquots of 150 µL of synthesized AgNPs were added to the first wells of the microplate from the stock nanoparticles solution (16 µM/mL). Serial dilutions were employed up to a final volume of 150 µL/well. Column 11 served as a positive control and was inoculated with culture medium and bacterial strain, and column 12 served as a negative control containing only culture medium. The microbial inoculums were adjusted spectrophotometrically to a concentration of 1.5 × 10^8^ CFU/mL and were inoculated in all the wells, except for the negative control (culture medium). The plates were incubated for 24 h at 37 °C for bacteria and 48 h at 28 °C for fungi. After the incubation period, the samples were analyzed visually (for color changing) and spectrophotometrically at a wavelength of 600 nm and 530 nm for bacteria and fungi, respectively. Absorbance reading was performed in an SpectraMax iD3 Multi-Mode Microplate Readers (San Jose, CA, USA). Assay was performed, in triplicate. The antimicrobial activity in the micro-broth dilution method was expressed in terms of the percent (%) of inhibition according to the following formula:

% inhibition = [(OD _positive control_ − OD _sample_)/(OD _positive control_ − OD _negative control−medium_)] × 100 (2)

where, OD = Optical density at 600 nm and 530 nm, for bacteria and fungi, respectively. Positive control is considered the pure culture of bacterial and fungal strains. The findings indicated that the activities of biogenic AgNPs tested against selected microbes were dose-dependent.

### 2.6. Statistical Analysis

All determinations regarding antimicrobial activity were performed in triplicate. The results were presented as the average value with the corresponding standard error.

## 3. Results

### 3.1. Biosynthesis of AgNPs Visual Inspection

**Figure 1:**
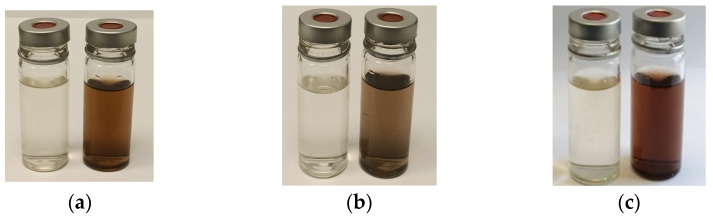
Visual evidence of color changing in a mixture of mycelium extract and silver salt, indicating the formation of AgNPs from 0 (left) to 72 h (right). (**a**) *P. lilacinum*; (**b**) *C. cladosporoides*; (**c**) *P. chrysogenum*.

Bioreduction of positive silver ions in aqueous solution can be observed by the change of initial color from pale yellow to reddish brown color, depending on the fungal specie (Figure 1).

### 3.2. UV-Vis Spectra

**Figure 2:**
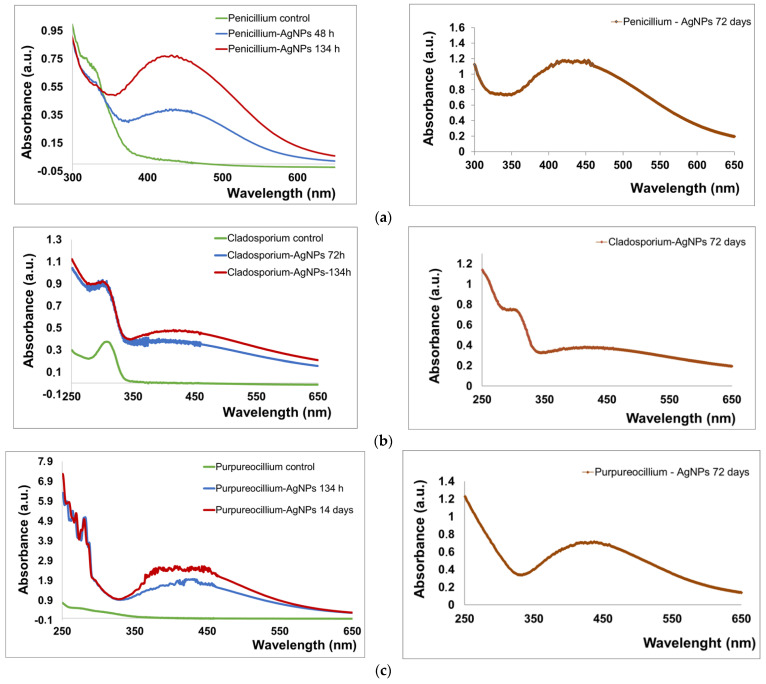
UV-Vis spectra of the biogenic silver nanoparticles mediated by fungal species. (**a**) *P. chrysogenum*; (**b**) *C. cladosporoides*; (**c**) *P. lilacinum.* On the right—UV-Vis spectra recorded after 72 days from the last data analysis, written in graphics from left column.

UV-Vis spectra confirming the biosynthesis of nanoparticles were recorded at several time intervals, for example 48 h, 72 h, 96 h, 120 h, and 134 h, even after 15, 20 or 25 days, and before 48 h there was no absorption peak recorded for any strain (Figure 2). To highlight the absorbance peaks, only data recorded at significant time points for each fungal strain are presented, considering that for many shapes the graphs would be difficult to be analyzed. For *P. lilacinum*, the specific plasmonic band appears to be symmetric from the first recordings (134 h), while for *Cladosporium* and *Penicillium*, an asymmetric shape can be observed due to the presence of aggregates particles. The wavelength value at which the peak was recorded for each strain was stable, and no further change in position was observed. The absorbance value increased after several hours of incubation. Also, the UV-Vis spectra of the AgNPs were recorded 72 days after the last data analysis mentioned in Figure 2. The peaks’ positions were kept at the same wavelength values, 440 nm for *Cladosporium*, 430–440 nm for *Penicillium*, and 430 nm for *Purpureocillium* nanoparticles, respectively.

### 3.3. SEM Analysis

**Figure 3:**
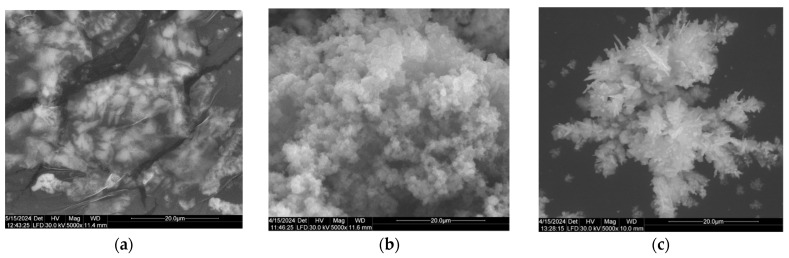
Scanning electron microscopy (SEM) images of AgNPs biosynthesized by filamentous fungi. (**a**) *P. chrysogenum*; (**b**) *C. cladosporoides*; (**c**) *P. lilacinum*.

Figure 3 depicts the appearance, shape, and morphological characteristics of AgNPs biosynthesized by the investigated fungal species.

The AgNPs obtained from *P. chrysogenum* are found to be a self-assembled mixture of triangular nanoparticles, some of which are pyramidal in shape and others are spherical in nature (Figure 3a). In this case, AgNPs tended to aggregate into small clusters, eventually forming larger particles. In Figure 3c, spherical, uniform-shaped and self-assembled silver nanoparticles AgNPs biosynthesized with *P. lilacinum* mycelium extract can be observed.

### 3.4. FTIR Spectra

**Figure 4:**
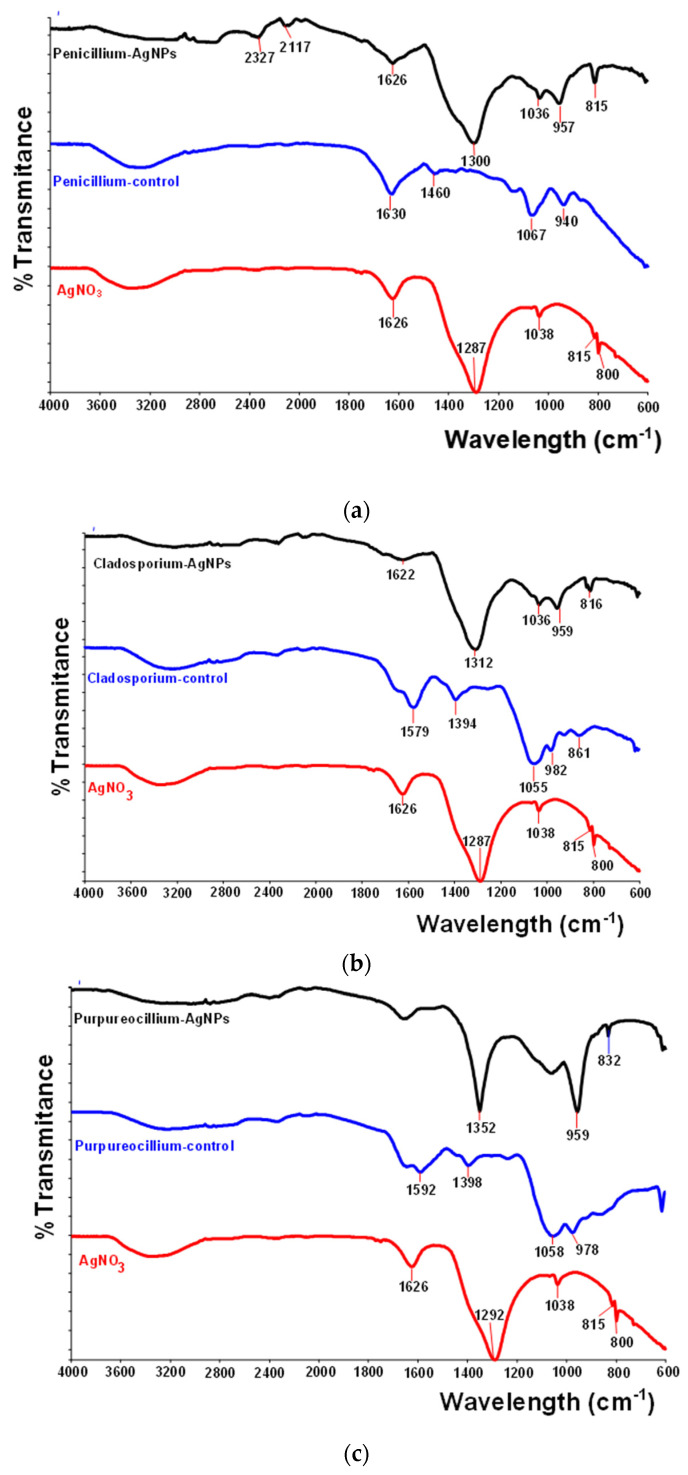
Fourier transform infrared (FTIR) spectroscopy profile for AgNPs with (**a**) *P. chrysogenum*; (**b**) *C. cladosporoides*; (**c**) *P. lilacinum*.

The functional groups of AgNPs were identified based on FTIR analysis. Therefore, the spectrum of silver nitrate (Figure 4a) had several peaks at 1625.91 cm^−1^. The high intensities were at 1287.08 cm^−1^, due to the stretching vibration of the N=O; 1037.54 cm^−1^; 814.54 (assigned to silver); and 800.47 cm^−1^. The corresponding peaks either shifted to higher values in the biosynthesized nanoparticles (assessed as *Penicillium*-AgNPs, Figure 3a), such as at 1626.01 cm^−1^, 1299.75 cm^−1^ (vibration of the N=O from AgNO_3_), 1043.50 cm^−1^, 802.57 cm^−1^, and 732.85 cm^−1^, or remained constant, such as 1036.14 cm^−1^, and 814.99 cm^−1^.

The spectrum of *P. chrysogenum*-control (aqueous extract of fungal mycelium) presented in the main absorbance bands: 1630.25 cm^−1^ was due to either C=C stretching of the alkene or N–H bending of the amine group; 1459.59 cm^−1^ assigned to C–O–C aromatic ether, phenolic C–O, and C–O–O–C ester stretch; 1067.23 cm^−1^ attributed to O–H bending in carboxylic acids; 949.51 cm^−1^ attributed to C–H bending in the aromatic ring (675–1000 cm^−1^). The band in the *P. chrysogenum*-control spectrum at 1630.25 cm^−1^ was modified in the synthesized silver nanoparticles and corresponded to an intense band in the FTIR spectrum of the silver nanoparticles at 1625.74 cm^−1^.

Likewise, the other fungi tested showed significant bands at more or less the same wavelengths as the Penicillium nanoparticles. Hence, the N=O vibration originating from AgNO3 appeared at 1300 cm^−1^ for *Penicillium*, at 1310 cm^−1^ for *Cladosporium*, and 1352 cm^−1^ for *Purpureocillium*, respectively. These bands proved the formation of silver nanoparticles by green synthesis based on the metabolites secreted by the fungal species in the culture medium. It is also important to highlight the peaks at 1350–1500 cm^−1^ which suggested the presence of aromatic and/or phenolic compounds in the fungal extract, for each strain being a certain position of the absorption band, as 1459.59 cm^−1^ for *P. chrysogenum* 1394 cm^−1^ for *C. cladosporoides*, and 1398.01 cm^−1^ for *P. lilacinum*. It should be mentioned that in all the spectra, a peak can be observed at a wavelength of around 2110 cm^−1^ (2116, 2117, 2108 etc.), which was signified by the isocyanate moiety secreted by the cells of the microorganism (–N=C=O).

### 3.5. DLS and Zeta Potential

**Figure 5:**
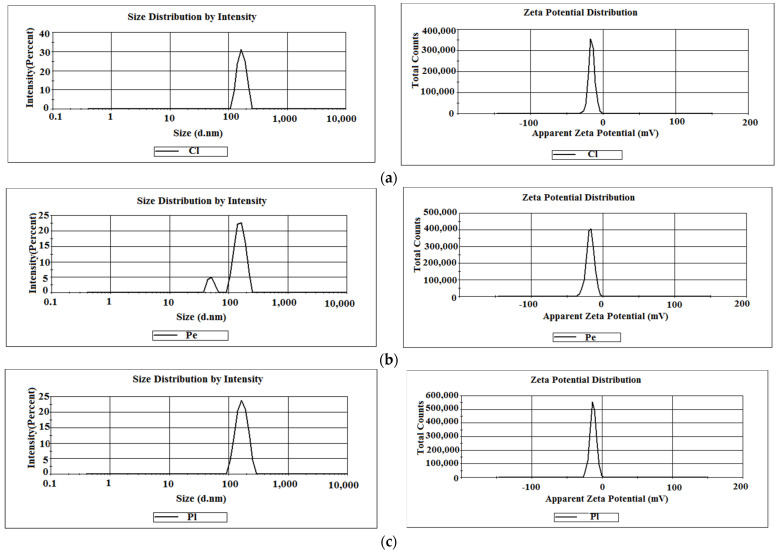

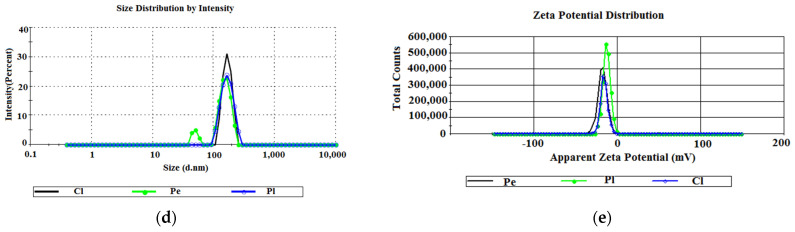
DLS and zeta potential of AgNPs biosynthesized with metabolites from fungal species. (**a**) *C. cladosporoides*; (**b**) *P. chrysogenum*; (**c**) *P. lilacinum*; (**d**) Overlap of size distribution by intensity; (**e**) Overlap of zeta potential distribution.

The particle size distribution (DLS) exhibited by the biosynthesized AgNPs in the colloidal phase was determined with the DLS technique (Figure 5). The size distribution was determined by intensity. The poly-dispersity index (PdI) is a measure of the heterogeneity of a sample based on size, reflecting the molecular mass distribution in a sample.

The diagrams indicated the following data for AgNPs: 168.3 nm (hydrodynamic diameter), Z-average = 165.6 nm, and PdI = 0.074 for *Cladosporium* with a single, narrow peak and clear distribution; 156.5 nm (intensity = 88.5%) for the majority of particles and a small shoulder containing nanoparticles with a hydrodynamic diameter of 49.76 nm (intensity = 11.5%), Z-average = 124.4 nm and PdI = 0.163 for *Penicillium*; 168.3 nm; and Z-average = 166.7 nm, and PdI = 0.279 for nanoparticles from *Purpureocillium* (Figure 5).

Zeta potential recorded for the biosynthesized AgNPs in the colloidal phase had the following negative values: −15.7 mV for *Cladosporium*; −17.8 mV for *Penicillium*; and −13.0 mV for nanoparticles from *Purpureocillium*.

### 3.6. TEM Analysis

**Figure 6:**
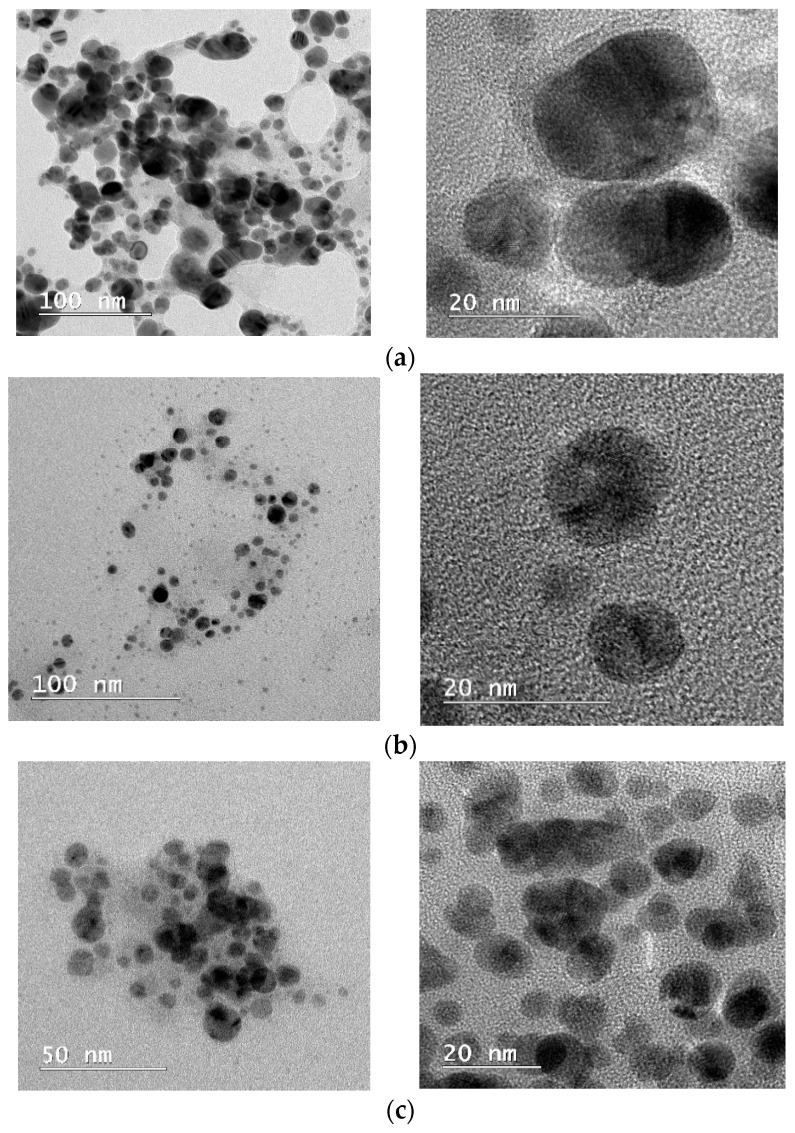
TEM images of AgNPs biosynthesized by filamentous fungi. (**a**) *Penicillium chrysogenum*; (**b**) *Cladosporium cladosporoides*; (**c**) *Purpureocillium lilacinum*.

TEM data provided insightful information about the morphology and size of the biogenic AgNPs (Figure 6). TEM analysis showed that the AgNPs produced with the aqueous mycelia extract of *Cladosporium*, *Penicillium*, and *Purpureocillium* were mostly spherical in shape with different sizes, with a maximum size of 20 nm.

### 3.7. Antimicrobial Study

**Figure 7:**
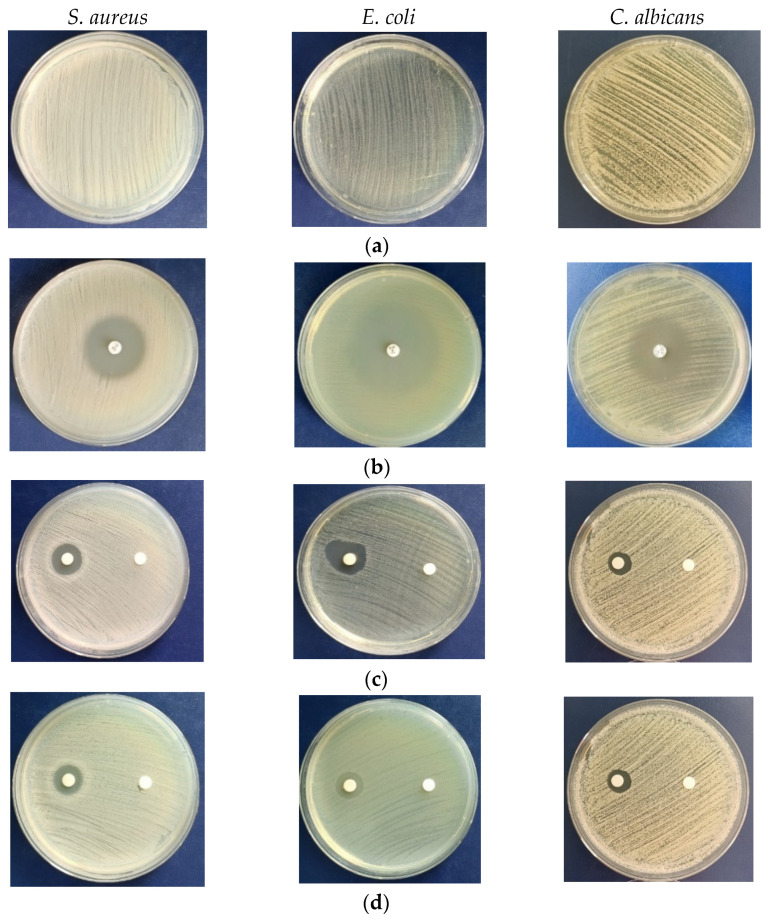

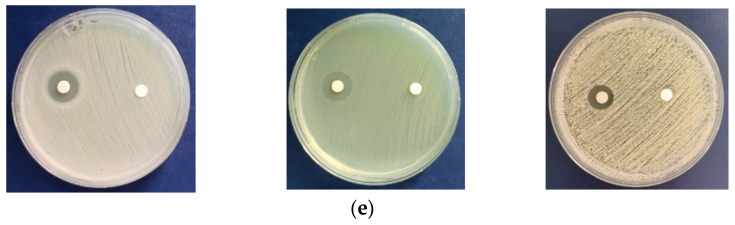
Antimicrobial activity of AgNPs biosynthesized by fungal species expressed versus *S. aureus*, *E. coli* and *C. albicans*. Zones of growth inhibition produced by AgNPs’ suspension in Petri plates with Muller–Hinton medium for bacteria and Sabouraud medium for yeast–fungi. (**a**) Images of pure cultures from control microbial strains. (**b**) Positive control for *S. aureus* (clindamycin, 2 µg), *E. coli* (norfloxacin, 10 µg), and *C. albicans* (ketoconazole, 50 µg). (**c**) Effect of AgNPs (left) and mycelium extract (right) from *C. cladosporoides.* (**d**) Effect of AgNPs (left) and mycelium extract (right) from *Penicillium chrysogenum*. (**e**) Effect of AgNPs (left) and mycelium extract (right) from *Purpureocillium lilacinum*.

In the current study, the antimicrobial activity of biogenic AgNPs was measured, as shown in Figure 7 and Table 1 and Table 2, with several microbial strains used as control. The maximum inhibition zones were obtained with biogenic AgNPs from *C. cladosporoides*. The diameter values decreased in the following order, as 22.50 ± 0.50 mm expressed against *S. aureus*: >20.83 ± 1.80 mm for *E. coli*; and >16.00 ± 1.00 mm for *C. albicans* (Table 1). On the second position was the activity of AgNPs from *P. lilacinum*, and the last was AgNPs from *P. chrysogenum.* For *S. aureus*, the inhibitions expressed by *Purpureocillium* and *Penicillium* were 3.7%, and 13.5% lower than *Cladosporium*, respectively. In *E. coli*, the inhibitions recorded for *Purpureocillium* and *Penicillium* were 0.82% and 7.76% lower, compared to the maximum values of *Cladosporium* AgNPs. In the *C. albicans* test, inhibition values were lower, with 13.63% and 7.13% for *Purpureocillium* and *Penicillium*, respectively, compared to the *Cladosporium* values.

The values of the diameters for the inhibition zone, expressed by the specific antibiotic and antifungal, were as follows: 23 mm for clindamycin (2 µg); 31 mm for norfloxacin; and 22 for ketoconazole (50 µg). No inhibition of control microbial strains were exhibited by mycelium extracts (Figure 7).

The growth of *S. aureus* was inhibited in the highest percentages by silver nanoparticles as follows: 93.75% for *C. cladosporoides*; 82.62% for *P. chrysogenum*; and 90.25% for *Purpureocillium lilacinum* (Table 2). *E. coli* and *C. albicans* were relatively more resistant to the activity of AgNPs, with inhibition values in the range of 62–69%.

**Figure 8:**
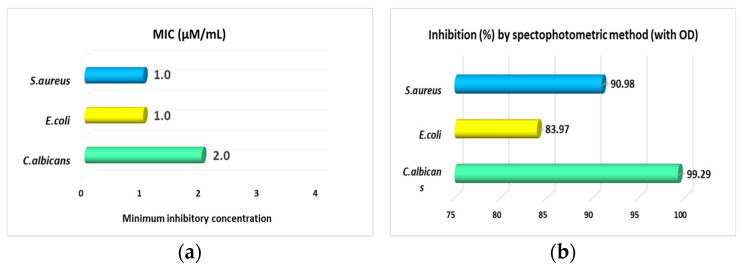
Minimal inhibitory concentration values of AgNPs obtained from *C. cladosporoides* against *S. aureus*, *E. coli* and *C. albicans*. (**a**) Graphic representation of MIC. (**b**) Graphic representation of microorganism’s inhibition (%).

The antimicrobial activity carried out in Petri plates was a preliminary test, requiring further evaluation of AgNPs for the determination of minimum inhibitory concentrations (MIC). MIC was determined only for AgNPs obtained from strain *C. cladosporoides* that expressed the highest antimicrobial activity (Figure 7 and Table 1). The AgNPs from *C. cladosporoides* showed the following MIC values: versus *S. aureus*, MIC = 1.0 μg/mL; versus *E. coli*, MIC = 1.0 μg/mL; and versus *C. albicans*, MIC = 2.0 μg/mL (Figure 8).

Thus, AgNPs from *C. cladosporoides* determined 90.98% of the inhibition of *S aureus* growth with 1.0 µM/mL MIC, while nanoparticles from *C. albicans* produced 99.29% inhibition with a double MIC concentration, of 2.0 µM/mL of nanoparticles.

## 4. Discussion

Recently, we have reported the ability of *Ganoderma lucidum* to synthesize silver nanoparticles through an environmentally friendly and pollution-free approach [49].

Considering the growing demand for new antimicrobial agents to overcome microbial resistance to existing treatments, this study represents a continuation of our ongoing work on the green synthesis of metal nanoparticles using filamentous fungi.

Filamentous fungi are a rich source of secondary metabolites with a significant role in the synthesis of nanoparticles. Diverse groups of fungi produce different proteins and secondary metabolites, which possess various biotechnological and pharmaceutical applications. Secondary metabolites are bioactive molecules with a low molecular mass, and they are chemically different, mainly composed of polyketides, alkaloids, terpenoids, and small peptides derived from amino acids. They are involved in the development of fungi and in interactions with other organisms. Due to these secondary metabolites, they have been widely used as biocontrol agents, allowing the management of plant diseases through a variety of mechanisms. Likewise, secondary metabolites play a role in the biosynthesis of nanoparticles. The residues of proteins and amino acids from biomolecules can bind to metals, covering the nanoparticles’ surface and protecting them as capping agents, limiting the particles aggregation, as well as the release of harmful substances. The direct connection between biocontrol activity and the formation of nanoparticles is very well documented, namely that the fungal species active in biocontrol of plant diseases can synthesize metal nanoparticles [43].

The experiments for green synthesis of AgNPs were conducted with certain fungal strains from our microbial collection chosen for different reasons, namely, the manifestation of a strong antagonism towards microorganisms, or, their notoriety according to the literature data analysis. Thus, we have demonstrated that *Cladosporium* [50] and *Purpureocillium*, former known as *Paecilomyces* [51], isolate and inhibit the growth and proliferation of phytopathogens through the secretion of various secondary metabolites that could be involved in the biosynthesis of nanoparticles. From the *Cladosporium* species, there were about 244 chemically defined compounds belonging to different classes of secondary metabolites extracted, such as azaphilones, benzofluoranthenones, coumarins and isocumarins, lactones, naphthalenones, macrolides, perylenequinones, sterols, and others [52]. *Cladosporium* secretes gliotoxin that acts as a capping and reducing agent because its oxygen and sulphur, in the carboxyl and hydroxyl groups and dithiol groups, respectively, bind to the positive charge of the nanoparticle surface [53,54]. According to several scientific reports [39,40,55], *Penicillium* seems to be a good candidate for the synthesis of silver nanoparticles, and we also tested our isolates. The *Penicillium* species produced secondary metabolites identified as belonging to diketopiperazine, benzodiazepine, quinoline alkaloids, clavine ergot alkaloids, polycyclic indole alkaloids, amino acid derivatives, polyketides, and terpenes (andrastins and phomenone) [56].

The criterion for the selection of the potential pathogenic microorganisms was based on their aggressiveness and notoriety. Many diseases and infections are caused by bacteria whose resistance to treatments has increased significantly. Thereby, the tests were carried out with relevant microorganisms, such as *Staphylococcus aureus*, *Escherichia coli*, and *Candida albicans.* Thus, *S. aureus* is the main pathogen in hospital and community infection, characterized by a high versatility, developing considerable microbial resistance towards antimicrobial agents [57]. *Escherichia coli* is used as a model for bacterium study exhibiting many strategies to infect the hosts. These species comprise non-pathogenic bacteria acting as commensals and belonging to the normal intestinal microbiota of humans, and also, pathogenic variants producing many diarrheal illnesses [58]. *Candida albicans*, the most common agent responsible for mucosal infections and systemic infection, induces over 90% of candidemia cases. *C. albicans* colonies proliferate rapidly and increase their virulence, causing severe infectious diseases, especially in immunocompromised patients [59].

Usually, the first step in proving the formation of nanoparticles is the color change of the reaction mixture consisting of an aqueous fungal extract and a metal salt precursor. All tested strains have demonstrated this behavior, and the intensity of color depends on the fungal specie.

The biosynthesis was studied through UV-Vis spectroscopy, a facile and indispensable technique, to investigate the nanoparticles synthesis. The resonant oscillation of electrons from the nanoparticles surface resulted in the formation of the surface plasmon resonance, which is the main characteristic of metal nanoparticles formation. In the current study, the synthesis of silver nanoparticles with metabolites from fungal species was confirmed by UV-Vis spectra, evidenced by the absorption peaks in the well documented region, similar with other reports [21,31,35,37,38,42,60,61,62]. The nanoparticles biosynthesis process was very slow compared to other studies where shorter times were recorded, e.g., 30 min at *Cladosporium* [38], 12 h at [63] and at *Penicillium oxalicum* [40]. In our experiments, we have obtained a single band in the UV-VIS spectra for *Cladosporium* (440 nm), *Penicillium* (438 nm), and *Purpureocillium* (like a plateau with maximum at 430 nm), respectively. For *Purpureocillium lilacinum* the presence of oscillations can be attributed to the aggregation properties [35,64]. It was demonstrated that a single SPR band is the result of spherical metal nanoparticles, while anisotropic particles gave two or more bands depending upon the shape of the nanoparticles [62]. It was observed that the silver nanoparticles solution was very stable, as demonstrated by UV-Vis measurements after more than two months. The peaks for each fungal strain were maintained at the same wavelength value and no evidence of nanoparticle flocculation was observed (Figure 2).

The morphology and distribution of the synthesized silver nanoparticles were studied using surface electron microscopy (SEM) that uses a high-energy electron beam to determine the surface’s topology. SEM analysis is widely used, due to it being non-destructive and allowing the repeated visualization of the same sample. In our work, the SEM images at different magnifications (1000× or 5000×) showed nanoparticles in relatively varied forms (spherical, triangular, pyramidal shaped) depending on the microbial source. To a certain extent, these results are consistent with the findings of other reports. Thus, AgNPs synthesized using *Cladosporium cladosporioides* were found to be spherically shaped, monodispersed, and no agglomeration points were observed [38]. The biomass from *Cladosporium halotolerans* was used to synthesize spherical, uniformly shaped, and well distributed silver nanoparticles [63]. Most of the nanoparticles synthesized using *Paecilomyces variotii* appeared round and slightly elongated [42]. In another study, SEM images showed spherical, homogenous, and well-dispersed AgNPs synthesized with metabolites from *Cladosporium oxysporum* [59].

Fourier transform infrared spectroscopy evidences the types of chemical interactions in molecules, producing a spectrum as a molecular fingerprint. It is well-documented that in aqueous or methanolic extracts from various species of fungi, FTIR spectra demonstrate the presence of aromatic rings, alkenes, aliphatic fluoro compounds, alcohols, ethers, carboxylic acids, esters, nitro compounds, aldehydes, ketones, alkanes, hydrogen-bonded alcohols, and phenols [64,65]. These functional groups identified by FTIR indicated the presence of several molecules of metabolically produced compounds that acted as capping agents during nanoparticles biosynthesis, reducing and preventing their agglomeration.

In the current study, FTIR spectra confirmed the following peaks for all fungal nanoparticles: the vibration of the N=O originated from AgNO_3_, located in domain 1299–1351 cm^−1^; the peaks at 1620–1650 cm^−1^ due to the C=C stretching of the alkene or the N–H bending of the amine group; the peaks at 1350–1500 cm^−1^ suggested the presence of aromatic and/or phenolic compounds in the fungal extract; and the peaks around 2110 cm^−1^ (2116, 2117, 2108, etc.,) signified by isocyanate moiety secreted by microorganism cells (–N=C=O). The shifts in the peaks as compared to mycelium aqueous extracts revealed that organic components such as alkene, nitro, amine, aromatic ester, and alcohols promoted the synthesis of silver nanoparticles during the reduction process of Ag+ to Ag(0). Also, it can be observed the broad adsorption band at 3300–3500 cm^−1^ suggested O–H stretching vibrations of benzene or phenols.

The positions of these bands are close to those reported in the recorded literature about nanoparticles biosynthesized by other fungal species. Thus, in a study dedicated to endophytic fungus *Penicillium polonicum* PG21, FTIR analysis highlighted the functional groups involved in the synthesis of AgNPs, at 1400 and 1550 cm^−1^, which indicated the presence of N–H aromatic secondary amine, attributed to the N–H stretching in the synthesized AgNP. It also highlighted the functional groups involved in string band at 1600–1650 cm^−1^, attributed to the carbonyl stretching in the proteins, suggesting the presence of proteins or other compounds on the AgNP surface, and contributing to stability and preventing agglomeration [60]. FTIR studies have shown the role of proteins, enzymes, and polyphenols from *Cladosporium cladosporioides* extracts in green synthesis of AgNPs [38]. In the FTIR spectrum of AgNPs biosynthesized with fungal extract from *Cladosporium pini-ponderosae*, several chemical bounds, C–O (ether), C–N (aromatic), C=C (aromatic), O–H (chelate), and N–H (secondary amine), were identified [11]. The biosynthesis of AgNPs using *Trichoderma longibrachiatum* were evidenced in the FTIR spectrum in the presence of the OH group of phenols, C–C stretching vibration modes in alkyne groups, N–H bending vibration of amine groups, symmetric–CH_3_ deformation in aromatic and aliphatic compounds and the –C–O stretch of alcohols, carboxylic acids, and esters, respectively [66].

DLS analysis was performed to determine the average size and the size distribution of particles in a suspension. The technique is commonly used for nanoparticles analysis, providing information on the size and state of aggregation of nanoparticles in a solution. The DLS diagrams indicated the close values of the average particle diameters, ofc165.6, 166.7 nm, for *Cladosporium*, and *Purpureocillium*, respectively, and the lower values of 124.4 nm for *Penicillium* nanoparticles. Also, the narrow peaks for *Cladosporium* and *Purpureocillium* can be seen from diagrams, in contrast with *Penicillium*, which presented a small shoulder at its lowest values of 4.76 nm. This aspect is important for future applications since a narrow peak indicates monodisperse nanoparticles, while a broad peak implies the polydispersity of nanoparticles [67]. A difference between the PdI values recorded for biosynthesized AgNPs was evidenced, with the highest PdI value being found to be 0.279 for the AgNPs obtained from *Purpureocillium*, while the lowest value of 0.074 was attributed to nanoparticles from *Cladosporium*. The PdI values lower than 0.05 indicate monodispersed particles and are specific to monodisperse samples, while values higher than 0.7 are common to a polydisperse distribution of particles [68]. In the current study, the reported PdI value of 0.074, showing a slightly polydisperse distribution, was very good and recommends *Cladosporium cladosporoides* for the green synthesis of metal nanoparticles.

Zeta potential studies offer information on the stability of nanoparticle suspensions. Thus, in the current study, all biosynthesized AgNPs had negative zeta values (−15.7 mV for *Cladosporium*, −17.8 mV for *Penicillium*, and −13.0 mV for *Purpureocillium)*, showing a good stability of nanoparticles. Also, the negative values of zeta potential indicated that silver nanoparticles are covered by negatively charged biomolecules, this aspect having a beneficial effect on stability, by reducing the repulsion between particles and the charged nanoparticles interacting with other molecules and ions [67,68,69]. It should be emphasized that no stabilizers were used in this study, which means that the secreted fungal metabolites acted not only as reducing agents but also for nanoparticles stabilization.

Similar results were described in other reports. AgNPs obtained from *Fusarium nygamai* presented a negative zeta potential of −10.79 mV [70]. Higher negative values of −41.9 mV and −44.2 mV were obtained for AgNPs biosynthesized with *Cladosporium cladosporioides* [71], and *Rubus discolor* [72], respectively.

The data obtained from TEM micrographs of AgNPs biosynthesized by our fungal strains showed mostly spherical particles without significant agglomeration, with diameter size less than 20 nm. Similarly, several other works have reported silver nanoparticles synthesized by fungal extracts [11,39,60]. TEM morphological and size analyses of AgNPs from *Penicillium oxalicum* revealed an average size of 6 nm. Also, another study showed for *Penicillium* nanoparticles a size range of 5.19–21.3 nm [40]. The average size range of silver nanoparticles obtained from *Cladosporium oxysporum* was found to be 5–8 nm [59].

The possible discrepancies between DLS and TEM are the consequence of the different specific approaches of the two mentioned analyses. DLS is a cumulative analysis, measuring the size of aggregates and not individual particles, while TEM is a local analysis that may capture some part of the aggregate more than others. Thus, the sizes obtained from DLS data are larger than TEM sizes, reflecting the true state of particles in a medium [73].

The evaluation of antimicrobial activity for biosynthesized AgNPs against several potential pathogenic microorganisms is the main target for future application in the health domain. The antimicrobial effectiveness of silver nanoparticles has been assessed for controlling bacteria, including Gram-positive *Staphylococcus aureus*, Gram-negative *Escherichia coli*, and the yeast fungi *Candida albicans*. According to experimental results, *S. aureus* was the most sensitive to biogenic AgNPs action, followed by *E. coli* and *C. albicans*, with their inhibition percentages being almost similar. Regarding the comparison between the activity of biogenic AgNPs from fungal sources, the most active against them was *C. cladosporoides*, followed by *P. lilacinum*, for which the values are almost equal to those obtained from AgNPs from *Cladosporium* extract. The antibiotic activity of the AgNPs was higher than that of AgNO_3_ solution or aqueous fungal extract alone. The higher sensitivity of prokaryotes (*S. aureus* and *E. coli*) than eukaryotes (*C. albicans*) to AgNPs inhibition was confirmed by another study carried out with biogenic AgNPs from *Aspergillus flavus* F5 [36].

Additionally, minimum inhibitory concentration (MIC) values were determined only for AgNPs obtained from *C. cladosporoides.* The spectrophotometric method for determining the inhibition of the tested microorganisms based on OD values confirmed the results obtained in the tests carried out with the gel diffusion method, with AgNPs being most active against *S. aureus*.

The inhibitory mechanism of biogenic AgNPs is very complex, and despite many studies focused on this topic, the exact mode of action has not been fully understood. According to several reports [21,30,74,75], the antibacterial activity exhibited by AgNPs depends on several parameters, such as shape, size, pH, temperature, and the capping agent used. Moreover, biogenic AgNPs have a series of interactions with different cellular components, through adhesion, osmosis, cell signal regulation and chemical interactions. In the cases of both Gram-positive and Gram-negative bacteria, the generation of reactive oxygen species (ROS), such as radical OH and hydrogen peroxide, and their interaction for antibacterial activity were evidenced. Since the morphologies of biogenic AgNPs obtained by microbial synthesis are various, it is difficult to determine a single mechanism of action. Depending on the genetic variability of the microbial species of the same genus and the strains of the same species, the metal nanoparticles that are synthesized from microbial sources may develop different degrees of antimicrobial activity [76].

The differences between the properties and behavior of biogenic silver nanoparticles, even in expressing antimicrobial activity, are the consequences of the specificity of each fungal specie, and the components extracted with water from mycelium.

## 5. Conclusions

In this study, we successfully synthesized silver nanoparticles (AgNPs) with metabolites from the filamentous fungi *Cladosporium cladosporoides*, *Penicillium chrysogenum*, and *Purpureocillium lilacinum*, using silver nitrate as a precursor. Through a range of analytical techniques, such as UV-Vis, SEM, TEM, FTIR, DLS, and zeta potential, the biogenic AgNPs were characterized, finding that those synthesized by *C. cladosporoides* exhibited the strongest antimicrobial effects against *S. aureus* (Gram-positive), *E. coli* (Gram-negative), and the yeast *C. albicans*.

Our results highlight the potential of *C. cladosporoides*-derived AgNPs as effective antimicrobial agents. Promising results were obtained against *S. aureus*, whose thick peptidoglycan layer typically presents a barrier to antibacterial agents. Moving forward, *C. cladosporoides* should be further investigated to optimize culture conditions, aiming to enhance the production of AgNPs with improved dispersity, stability, and biocompatibility for potential therapeutic applications.

## Figures and Tables

**Table 1:** Assessment of the inhibitory effect of biosynthesized AgNPs with fungal species on control microbial strains.

AgNPs Biosynthesized by Fungal Species	Diameter of Inhibition Zone (mm) *		
	*Staphylococcus* *aureus*	*Escherichia* *coli*	*Candida* *albicans*
*C. cladosporoides*	22.50 ± 0.50	20.83 ± 1.80	16.00 ± 1.00
*P. chrysogenum*	19.83 ± 0.20	19.33 ± 1.50	13.66 ± 0.20
*P. lilacinum*	21.66 ± 1.50	20.66 ± 2.00	14.08 ± 1.00

* Data represent the mean ± standard error of triplicate samples performed in the same experimental conditions.

**Table 2:** Inhibition percentage expressed by biogenic AgNPs.

AgNPs	Inhibition of Microorganisms Growth (%)		
	*S. aureus*	*E. coli*	*C. albicans*
*C. cladosporoides*	93.75	67.20	69.56
*P. chrysogenum*	82.62	62.35	62.10
*P. lilacinum*	90.25	66.65	64.00

## Data Availability

The original contributions presented in this study are included in the article. Further inquiries can be directed to the corresponding authors.
